# Unraveling the role of hypoxia-inducible factors in cutaneous melanoma: from mechanisms to therapeutic opportunities

**DOI:** 10.1186/s12964-025-02173-4

**Published:** 2025-04-09

**Authors:** Arianna Bellazzo, Barbara Montico, Roberto Guerrieri, Francesca Colizzi, Agostino Steffan, Jerry Polesel, Elisabetta Fratta

**Affiliations:** 1 Immunopathology and Cancer Biomarkers, Centro di Riferimento Oncologico di Aviano (CRO), IRCCS, via Franco Gallini, 2, Aviano, 33081 PN Italy; 2 Unit of Cancer Epidemiology, Centro di Riferimento Oncologico di Aviano (CRO), IRCCS, via Franco Gallini, 2, Aviano, 33081 PN Italy

**Keywords:** Cutaneous melanoma, Hypoxia-inducible factors, Hypoxia, Normoxia, Non-coding RNAs

## Abstract

Hypoxia is a common feature of solid malignancies, including cutaneous melanoma (CM). Hypoxia-inducible factor (HIF)-1α and HIF-2α orchestrate cellular responses to hypoxia and coordinate a transcriptional program that promote several aggressive features in CM, such as angiogenesis, epithelial-mesenchymal transition, metastasis formation, metabolic rewiring, and immune escape. BRAF^V600E^, which is the most frequent mutation observed in CM patients, usually increases HIF-α signaling not only in hypoxia, but also in normoxic CM cells, enabling HIF-1α and HIF-2α to continuously activate downstream molecular pathways. In this review, we aim to provide a comprehensive overview of the intricate role and regulation of HIF-1α and HIF-2α in CM, with a brief focus on the complex interactions between HIF-α subunits and non-coding RNAs. We also discuss HIF-α-mediated cellular responses in normoxia along with the mechanisms that allow HIF-α subunits to maintain their stability under normal oxygen conditions. Finally, we resume available evidence on potential therapeutic approaches aimed at targeting HIF-1α and/or HIF-2α.

**Supplementary Information:**

The online version contains supplementary material available at 10.1186/s12964-025-02173-4.

## Introduction

Oxygen is crucial for cell survival and function. Therefore, a prolonged reduction of oxygen availability (hypoxia) usually represents a lethal condition for cells and tissues. On the contrary, about 50–60% of solid tumors are commonly affected by hypoxia [1]. In fact, the rapid and uncontrolled growth of tumor cells commonly outstrips the oxygen supply from the preexisting blood vessels [2].

The adaptive response to hypoxia is mainly mediated by the hypoxia-inducible factors 1 (HIF-1), 2 (HIF-2), and 3 (HIF-3). HIF proteins are heterodimers composed of an oxygen sensitive α subunit (HIF-1α, -2α or -3α) and the HIF-1β subunit, which is constitutively expressed, independently from oxygen levels. In contrast, in normoxia, cytoplasmic α subunits show a very short half-life, since they are continuously degraded via the ubiquitin-proteasome system, whereas under hypoxic conditions, α subunits are stabilized via post-translational modifications and translocated into the nucleus, where they dimerize with HIF-1β [3].

**Fig. 1:**
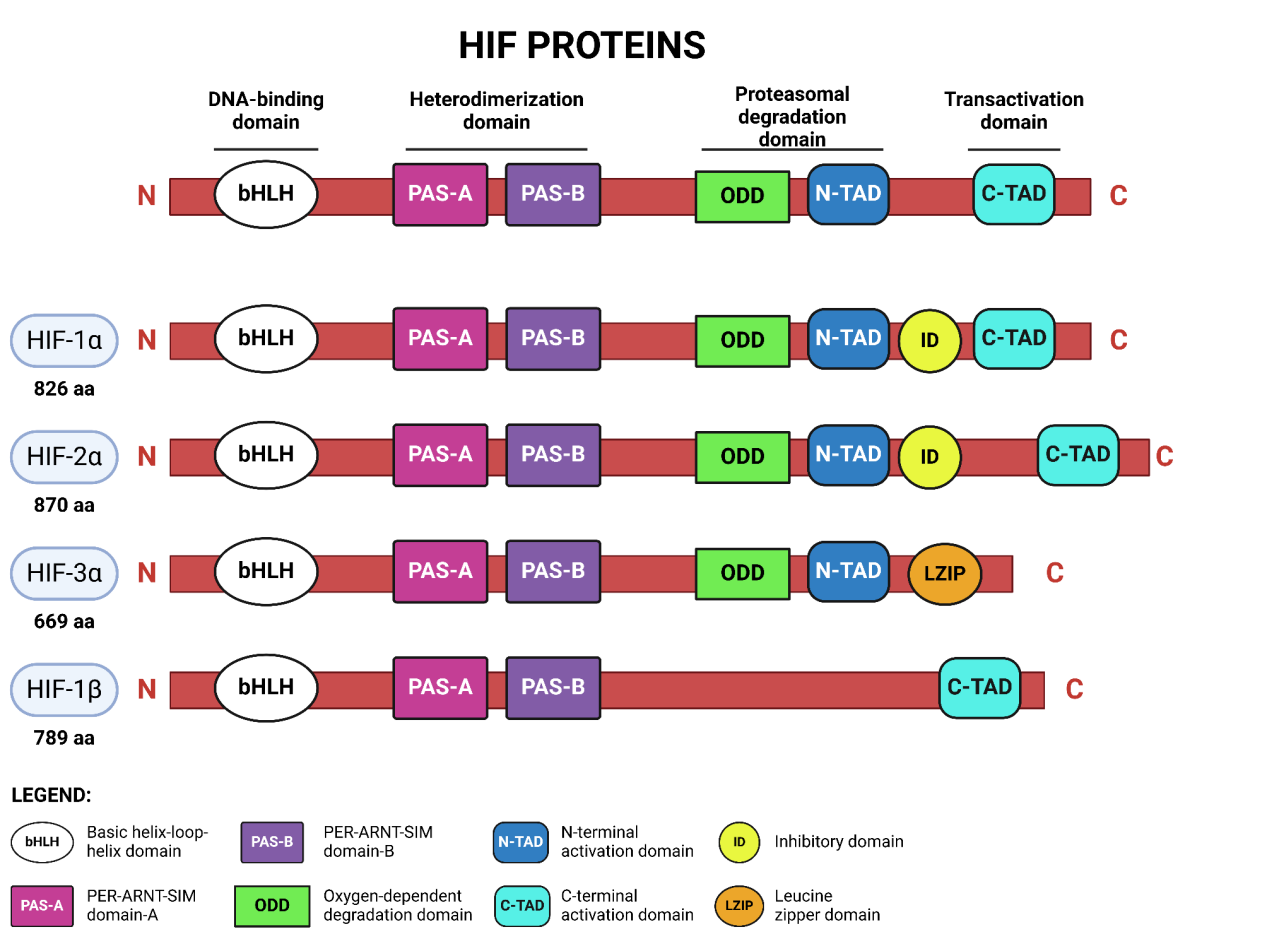
Schematic illustration of HIF-1α, HIF-2α, HIF-3α isoforms and HIF-1β protein. As shown, HIF-α proteins possess a bHLH and two PAS domains, responsible for the heterodimerization. Instead of having the C-TAD domain, HIF-3α has a LZIP domain that allows the protein interaction. The constitutively expressed HIF-1β does not contain the ODD, N-TAD and ID domains. Abbreviations are listed in the legend section. Created in BioRender (https://BioRender.com/s38c417)

HIF-1 was initially discovered through a study on the erythropoietin gene [4]. Subsequently, the structural analysis of the HIF-1α protein revealed four distinct domains: a basic helix-loop-helix (bHLH) domain for DNA binding and dimerization, two Per-ARNT-Sim (PAS) domains for dimerization and target gene specificity, an oxygen-dependent degradation (ODD) domain, and two transactivation domains (N-TAD and C-TAD) [5]. HIF-2α is closely related to HIF-1α, whereas HIF-3α misses the C-TAD domain, which is required for HIF-1α and HIF-2α transcriptional activity. In fact, the C-TAD was found to interact with CREB-binding protein (CBP) and p300 co-activators to activate gene transcription [6]. As shown in Fig. 1, α subunits are distinct from HIF-1β since they all possess the ODD domain, which is crucial for preventing their degradation in hypoxic condition [7].

The existence of three different α subunits evokes questions about target genes exclusively induced by each of them, as well as the specificity of their regulated phenotypes. Unlike HIF-1α and HIF-2α, that are widely studied and clearly linked to development and carcinogenesis, HIF-3α functions are still largely unknown, especially in the context of malignancies [8–11]. The presence of multiple transcript variants of HIF-3α, showing tissue-specific expression and potential divergent roles in the regulation of gene expression, makes harder to determine its molecular functions [12]. Differently, HIF-1α and HIF-2α are well characterized, with a list of both common and unique target genes, which reflect their similar as well as independent roles in different tumor types (for review see [13]). Importantly, N-TAD domain, that differs between the two isoforms, has been shown to contribute to HIF-1α and HIF-2α target genes specificity, whereas the C-TAD domain is homologous and promotes the expression of HIF-1/HIF-2 common target genes [14].

Knockout mice have showed that both HIF-1α and HIF-2α are not-redundant and essential in development, since the targeted independent disruption of both subunits resulted in embryonic lethality. Nevertheless, embryos died of different causes and at different age. For instance, HIF-1α -/- embryos did not survive beyond cardiac and vascular defects, whereas HIF-2α -/- had normal systemic vasculature but suffered of bradycardia, and did not exhibit completed lung maturation [15]. Accordingly, HIF-1α and HIF-2α pattern of expression vary among tissues. Specifically, HIF-1α is detected ubiquitously, whereas HIF-2α exhibits a more tissue-specific expression pattern [16]. In response to reduced oxygenation, HIF-1α primarily mediates acute responses, whereas the chronic hypoxic response involves HIF-2α activation [17]. Although both subunits have been delineated as key factors in promoting tumor progression, however, the specificity of their functions remains to be fully elucidated.

## Epidemiological characteristics and genetic alterations in cutaneous melanoma

Cutaneous melanoma (CM) represents a very aggressive neoplasm originating from the malignant transformation of melanocytes, accounting for about 20% of all cutaneous cancers [18]. Although it is the most lethal form of skin cancer, the 5-year relative survival is above 80%, with worse prognosis for nodular CM compared to lentigo maligna or superficial spreading CM [19]. CM incidence has dramatically increased in recent decades among light-skinned populations. Approximately 85% of CM occurring annually affect populations from North America, Europe and Oceania, with Australia and New Zealand showing the highest CM age-standardized incidence rates (40–50 cases/100000 per year) [18]. In Europe, the average incidence of CM is 9 cases/100,000 per year, with higher prevalence in Nordic populations (about 20 cases/100000 per year) [18].

Genetic alterations are common in both familial and sporadic CM, with frequently alteration of the MAPK/ERK signaling. In particular, the constitutive activation of the MAPK/ERK signaling caused by mutations within BRAF gene appears as a major driver of CM tumorigenic potential and survival [20, 21]. BRAF is a serine/threonine protein kinase encoded by the BRAF gene located on chromosome 7. At present, it is well known that over 90% of BRAF mutations occur in exon 15 and include substitutions of valine at position 600 (V600) with another aminoacid. In particular, the substitution of glutamic acid for valine (V600E, codon GTG > GAG), accounts for 70–80% of the BRAF^V600^ mutations. The second most common mutation is BRAF^V600K^ substituting lysine for valine (GTG > AAG), followed by BRAF^V600R^ (GTG > AGG), and by an infrequent two-nucleotide variation of the predominant mutation that includes the BRAF^V600^ ′E2′ (GTG > GAA) and the BRAF^V600D^ (GTG > GAT) [22]. BRAF mutations are able to produce an activation of the kinase domain of the protein, leading to an uncontrolled stimulation of cell proliferation [23]. NRAS is the second most frequent mutation in CM (15–20%), after BRAF [24]. In the majority of cases (> 80%), NRAS gene presents a missense mutation within codon 61, which disrupts the GTPase activity of NRAS, locking it in its active conformation, independent of upstream RTK activation [25]. Besides BRAF and NRAS, a small subset of CM presents NF1 (10–15%) and c-Kit (1–3%) mutations [25]. Finally, a high frequency of PTEN mutations have been reported in highly metastatic CM, where they frequently coexist with BRAF mutations, but not with NRAS [25].

Constitutive expression of HIF-1α and HIF-2α has been frequently detected in CM [26]. BRAF-mutant CM, in particular, usually exhibits dramatic activation of HIF-1α signaling, often uncoupled from hypoxia [27–29]. Along this line, microarray profiling on 30 cell lines derived from various stages of CM progression and 5 melanocytes showed that HIF-1α expression was significantly higher in BRAF-mutant CM cell lines respect to cells with wild-type BRAF or melanocytes. Accordingly, suppression of BRAF^V600E^ decreased HIF-1α expression and impaired CM cell survival and proliferation, whereas its overexpression increased hypoxic tolerance through HIF-1α up-regulation, suggesting that the oncogenic activity of BRAF^V600E^ might be partially mediated directly through the HIF-α signaling pathway [27].

In the last years, a better understanding of the CM biology has resulted in the development of different FDA approved therapeutic strategies relying on the use of small molecule inhibitors of BRAF (BRAFi) and MEK (MEKi) and blockers of the immune checkpoint molecules. The advent of targeted and immune-based therapies has not only expanded the treatment options for CM but also proven clinical benefits in patients with unresectable or metastatic CM. However, primary and acquired resistance often limit the clinical effectiveness of these therapies. Importantly, aberrant expression of HIF-1α and HIF-2α might influence response to therapy and impact on drug resistance in CM.

Hence, this review aims to provide an overview of the activity and the regulation of HIF-1α and HIF-2α in CM, not only under hypoxia, which occurs when oxygen (O_2_) levels fall below 1 kPa, but also in normoxic environments. With the term “normoxia”, we refer to the atmospheric oxygen (O_2_) levels of 18 kPa at which the majority of pre-clinical studies with cells are conducted. This O_2_ tension is clearly higher respect to that measured in physiological tissues (i.e. 3–8 kPa in the skin), and termed “physoxia” [30, 31]. Based on these considerations, a list of papers with the terms “CM”, “hypoxia”, “normoxia” and/or “HIF” were retrieved and selected, mainly focusing on those published in the last two decades. The potential therapeutic targeting of HIFs to improve treatment efficacy in CM will be also discussed.

## HIF-1α-related targets in hypoxia and normoxia in CM

In CM, HIF-1α mediates the transcription of genes involved in several processes, including angiogenesis and vascular remodeling, epithelial to mesenchymal transition (EMT), metastasis, and cellular metabolism, in both hypoxic and normoxic conditions. A selection of the most frequently HIF-1α-target genes in CM is given in Table 1.

**Table 1:** HIF-1α and HIF-2α targets in CM

Pathway	HIF-α	Target gene (s)	Hypoxia/Normoxia	Cell line(s)	BRAF/NRAS mutational status	Reference
*Angiogenesis and vasculogenic mimicry*	HIF-1α	MET	Hypoxia	Hs29-4T	BRAF^V600E^	[38]
	HIF-2α	sVEGF	Hypoxia/normoxia	B16F10 A375	wt BRAF^V600E^	[64]
	HIF-1α/HIF-2α	VEGF-A VEGF-C	Hypoxia/normoxia	1007 M10 M14 SK-Mel-28	wt *n.a. BRAF^V600E^ BRAF^V600E^	[39]
*EMT and metastasis*	HIF-1α	Genes *downregulated* in the switch from a proliferative to an invasive phenotype *CAPN3* *DAPK1* *GALNT3* *GPM6B* *MITF* *MYO1D* *TNFRSF14* *TYRP1* Genes *upregulated* in the switch from a proliferative to an invasive phenotype *ADAM12* *AXL* *BIRC3* *COL13A1* *CRIM1* *FGF2* *FLNB* *HS3ST3A1* *KCNMA1* *ITGA3*	Hypoxia	M000921 M010817 M080307 M080423	BRAF^V600E^ NRAS^Q61R^ n.a. n.a.	[41]
	HIF-1α	HIF1α repressed *MITF* promoter through the transcription factor BHLHB2	Hypoxia	B16F10 SK-Mel-28	wt BRAF^V600E^	[42]
	HIF-1α	HIF1α induced a switch from ROR-1 to ROR-2 to activate Wnt5A signalling pathway	Hypoxia	G361 M93-047 UACC1273EV UACC647 UACC903 WM35 WM793 WM983B 451Lu 1205Lu	BRAF^V600E^ NRAS^Q61R^ n.a. BRAF^V600E^ BRAF^V600E^ BRAF^V600E^ BRAF^V600E^ BRAF^V600E^ BRAF^V600E^ BRAF^V600E^	[43]
		HIF1α directly bound to *CD147* promoter to induce MMP-2 activation	Hypoxia	A375 G361	BRAF^V600E^ BRAF^V600E^	[44]
	HIF-1α	*uPAR*	Hypoxia	R-18	n.a.	[48]
	HIF-1α	TB-4	Hypoxia	B16F10	wt	[49]
	HIF-1α	MET	Hypoxia	Hs29-4T	BRAF^V600E^	[38]
	HIF-1α	*BIRC-7*	Hypoxia/normoxia	A875 M14	wt BRAF^V600E^	[52]
	HIF-1α	*Rab27*	Normoxia	B16-F10	wt	[53]
	HIF-2α	Snail	Hypoxia	1205Lu WM35 WM793 WM115A WM3523A	BRAF^V600E^ BRAF^V600E^ BRAF^V600E^ BRAF^V600E^ BRAF^V600E^	[65]
	HIF-2α	AP2a	Hypoxia	501-mel	BRAF^V600E^	[66]
	HIF-1α/HIF-2α	CTGF	Hypoxia	K457	BRAF^V600E^	[67]
	HIF-1α	SFK via *PDGFRA* MT1-MMP	Hypoxia	A375SM WM266-4	BRAF^V600E^	[68]
	HIF-2α	SFK via *FAK* MMP-2				
*Metabolic reprogramming*	HIF-1α	*GLUT1* *HK2* *LDHA*	Hypoxia	SbCl2 WM35 WM239A WM1158 WM1232 WM1366 WM3211 451Lu 1205Lu Mel-Im Mel-Ju	NRAS^Q61R^ BRAF^V600E^ BRAF^V600E^ BRAF^V600E^ BRAF^V600E^ NRAS^Q61R^ wt BRAF^V600E^ BRAF^V600E^ n.a. n.a.	[56]
	HIF-1α	*PDK1*	Normoxia	Mel 272 Mel 593 Mel 611	BRAF^V600E^ BRAF^V600E^ BRAF^V600E^	
	HIF-1α	GLUT1 and LDHA overexpression ATP5ME, NDUFA6, NDUFB5, NDUFB6 and NDUFS8 downregulation	Hypoxia	A2058 HT-144 SK-Mel-5 SK-Mel-28	BRAF^V600E^ BRAF^V600E^ BRAF^V600E^ BRAF^V600E^	[57]
	HIF-1α	PKM2	Hypoxia	RPMI 8322 SK-Mel-23 SK-Mel-103 SK-Mel-187 VMM39 WM2664 501-mel 526-mel 624-mel	wt wt NRAS^Q61R^ wt NRAS^Q61R^ BRAF^V600E^ BRAF^V600E^ BRAF^V600E^ BRAF^V600E^	[58]
	HIF-1α	*PDK1*	Normoxia	A375 MEWO Sk-mel-28 WM35	BRAF^V600E^ wt BRAF^V600E^ BRAF^V600E^	[59]
	HIF-1α	GM3S	Hypoxia	B16F10 G361	wt BRAF^V600E^	[61]
*Inflammation and immunomodulation*	HIF-1α	*ACKR2*	Hypoxia	B16F10	wt	[63]
	HIF-1α /HIF-2α	Treg	Hypoxia/normoxia	B16F10	wt	[70]
	HIF-1α /HIF-2α	CD8 + T cells	Hypoxia	B16	wt	[71]

* Data not available

*Angiogenesis and vasculogenic mimicry.* It has become increasingly clear that the interaction of endothelial cells (EC) and cancer cells affects tumor growth and vascularization. Hypoxia and HIF-α signature are master regulators of microcirculation acquisition, thus supporting malignant growth and hematogenous dissemination. The delineation of molecular mechanisms of neo-angiogenesis in cancer has revealed a critical role for HIF-1α in positively regulating pro-angiogenic factors, including vascular endothelial growth factor (VEGF) and its receptors (i.e. FLK-1 and FTL-1), angiopoietins and platelet-derived growth factor beta [32]. To date, although the hypoxia-induced new vessel formation is a common feature of CM, the number of studies directly focused on HIF-1α and angiogenesis in CM progression and treatment are still limited. However, it has become clear that HIF-1α fosters pro-angiogenic factors expression in CM [33]. Indeed, Trisciuoglio et al. reported that Bcl-2, via BH4 domain, increased HIF-1α half-life by counteracting its ubiquitination, in order to enhance HIF-1α-mediated VEGF expression and secretion in CM cells exposed to hypoxia [34]. Of interest, vasculogenic mimicry, a process that cooperates with neo-angiogenesis for the recruitment of new vessels, was firstly identified in CM and uveal melanoma through in vitro and in vivo studies [35, 36]. In particular, vasculogenic mimicry describes the ability of CM cells to express endothelium-associated genes and ECM-remodeling proteins to mimic the presence and function of EC and to support a capillary-like structure formation [35, 36]. Mouse CM cells injected in mouse ischemic limbs increased the expression of HIF-1α and VEGF to support the formation of vasculogenic mimicry channels [37]. In a study by Comito et al., once stabilized upon hypoxia-induced accumulation of mitochondrial reactive oxygen species (ROS), HIF-1α activated MET expression in CM cells to promote capillary-like structures formation and vasculogenic mimicry [38]. In a subsequent study by Spinella et al., hypoxia induced production of endothelin-1 (ET-1) that, in turn, sustained HIF-1α/HIF-2α-mediated VEGF-A and VEGF-C expression in CM cells and EC, suggesting HIFs involvement in promoting endothelia remodeling and vasculogenic mimicry. Consistent with this hypothesis, silencing of HIF-1α/HIF-2α completely abrogated cell migration and hypoxia-induced capillary-like structure acquisition, in both CM and EC exposed to CM cells secretome. Furthermore, ET-1 capacity to increase VEGF-A and VEGF-C mRNA and protein expression in CM and EC was reduced [39].

*EMT and metastasis.* The expression of HIF transcription factors has been suggested to correlate with the increased malignant potential of melanocytes. In fact, several genes that act as key players in melanogenesis and melanocyte behavior contain putative functional hypoxia response elements (HREs) and, therefore, might be directly targeted by HIF transcription factors [26]. In another study, transcriptomic analysis of murine immortalized melanocyte cells exposed to hypoxia identified a signature that included thirteen EMT-associated genes, of which only two, *Plod1* and *Plod2*, exhibited HRE motifs within their promoters [40], supporting that HIF-1α regulation might occur through both direct and indirect mechanisms.

In hypoxic conditions, HIF-1α has been demonstrated to contribute to CM heterogeneity, and to trigger metastatic progression by reprogramming gene expression patterns in proliferative CM cells, making them more invasive in in vitro assays. The incubation of a set of human CM cells in hypoxia chamber allowed to identify eighteen HIF1α-dependent genes that were differentially regulated in the switch from a proliferative to an invasive phenotype in CM cell cultures. Interestingly, the microphthalmia-associated transcription factor (*MITF*) and several of its target (i.e. *TRP1*, *TNFRSF14*, *CAPN3*, *GPM6B*, and *DAPK1*) were among repressed genes [41], indicating that HIF1α-regulated *MITF* might act as a key regulator of the process of hypoxia-induced phenotype switching in CM. In accordance, a study by Cheli et al. revealed that, in hypoxic condition, HIF-1α positively regulated the expression of the transcription factor basic helix-loop-helix protein 2 (BHLHB2) to inhibit *MITF*, thus increasing the tumorigenic and metastatic properties of CM cells [42]. The interplay between HIF-1α and the receptor tyrosine kinase-like orphan receptor (ROR)-1 and ROR-2 has been intimately involved in the switch from a proliferative to an invasive phenotype as well. More specifically, following hypoxia exposure, Wnt5A stabilized HIF-1α, resulting in ROR-2 up-regulation and decreased expression of MITF and ROR-1. ROR2 expression associated with a more aggressive phenotype and was critical for Wnt5A-mediated invasion and metastasis in CM [43].

HIF-1α was also shown to orchestrate EMT and metastasis in CM by increasing the expression of proteins and enzymes directly implicated in extracellular-matrix (ECM) remodeling, including matrix metalloproteinase-2 (MMP-2) and urokinase-type plasminogen activator receptor (uPAR). Once activated by hypoxia, HIF-1α enhanced the invasion and metastatic potential of CM cells by fostering MMP-2 expression through the involvement of CD147 [44], a key inducer of MMPs [45]. Chromosome immunoprecipitation assay supported that HIF-1α directly bound to *CD147* promoter. Indeed, point mutations within *CD147* promoter attenuated MMP-2 response to hypoxia both in vitro and in vivo [44]. The interaction between urokinase-type plasminogen activator receptor (uPAR) and its ligand, which is mainly involved in pericellular proteolysis, is a central step in mesenchymal motility. By binding to the HRE within *uPAR* promoter, HIF-1α mediated *uPAR* transcription in various cancers under hypoxia [46, 47]. In CM, in particular, uPAR overexpression promoted spontaneous lymph nodes metastasis in in vivo models [48]. In addition to MMP-2 and uPAR, hypoxia might induce CM metastasis through a putative cooperation between HIF-1α and thymosin beta-4 (Tβ4) [49], an actin-sequestering molecule involved in cytoskeletal reorganization [50]. At present, the molecular mechanism by which the HIF-1α/Tβ4 axis contributes to CM metastasis is still largely unknown. Nevertheless, in a study by Makowiecka et al., Tβ4 was not only a component of focal adhesions, but also directly regulated their formation, thus altering the adhesion abilities of CM cells [51].

As described above, the ROS/HIF-1α/MET signaling axis has been strongly involved in hypoxia mediated vasculogenic mimicry in CM. In the same study, MET expression and activation were also correlated with enhanced CM cells migration in vitro and lung metastasis formation in vivo [38], thereby suggesting that HIF-1α and MET might cooperate in eliciting an escape strategy to avoid the hypoxic CM environment.

More recently, Xu et al. unveiled baculoviral IAP repeat-containing protein 7 (*BIRC7*) as a downstream HIF-1α target gene. Although BIRC7 is known to act as an apoptosis inhibitor, its knockdown inhibited the invasion of A875 and M14 CM cells in both hypoxia and normoxia [52], indicating that even in normoxic condition HIF-1α could sustain invasiveness. Under lactate stimulation, HIF-1α was found to drive lung metastasis in B16F10 transplanted mice, specifically by regulating Rab27 expression and extracellular vesicles secretion [53].

*Metabolic reprogramming*. HIF-1α represents the main transcription factor for the induction of genes encoding glucose transporters (GLUT 1/4) and glycolytic enzymes, such as aldolase, enolase, lactate dehydrogenase (LDHA) and phosphoglycerate kinase-1, allowing hypoxic tumors cells to take up glucose more efficiently. Additionally, HIF-1α can suppress oxidative phosphorylation (OXPHOS) by reducing metabolites entry into the tricarboxylic acid cycle (TCA), via the direct induction of the key glycolytic enzyme pyruvate dehydrogenase kinase (PDK) 1 that, in turn, phosphorylates the pyruvate dehydrogenase (PDH) complex, thus preventing the conversion of pyruvate into acetyl-CoA [54]. Respect to melanocytes, malignant CM cells, particularly those harboring the BRAF^V600E^ mutation, exhibit the Warburg effect with high glucose consumption and more lactate production even in the presence of oxygen and fully functioning mitochondria [55]. The BRAF^V600E^ mutation might transactivate a panel of transcriptional regulators of glycolysis, including HIF-1α [28] and its target genes *GLUT1*, hexokinase 2 (*HK2*), *LDHA* [56], and *PDK1* [29]. Under hypoxia, HIF-1α-dependent upregulation was accompanied by a decreased expression of several components of the OXPHOS, including ATP5ME, NDUFA6, NDUFB5, NDUFB6 and NDUFS8 [57]. HIF-1α activation could also up-regulate pyruvate kinase M2 (PKM2), a rate-limiting enzyme of glycolysis, further minimizing OXPHOS [58].

HIF-1α-dependent glycolytic program offers metabolic plasticity but also growth advantage for CM cells. For example, Liu et al. demonstrated that HIF-1α enhanced *PDK1* transcription by recruiting the Ku80 protein to *PDK1* promoter. Consistently, treatment with melatonin, which affects the stability of HIF-1α at the protein level, suppressed the binding of Ku80 to the *PDK1* promoter and inhibited CM growth [59]. By reprogramming the glucose metabolic pathway and ensuring scavenging of mitochondrial free radicals, HIF-1α enhances the antioxidant capacity of tumor cells, fostering radioresistance [60]. In this context, it has been demonstrated that HIF-1α could indirectly decrease the expression of the ganglioside GM3 synthase, thus reducing synthesis of glycosphingolipids and increasing resistance of CM cells to oxidative stress and radiation therapy [61].

*Inflammation.* In silico analysis suggested that inflammatory response, together with increased recruitment of T-cells, B-cells, NK-cells, monocytes and macrophages, were strongly associated with CM [62]. Intriguingly, HIF-1α has emerged as one the main pathways implicated in the regulation of the inflammatory response. Atypical chemokine receptor 2 (*ACKR2*), which regulates a number of pro-inflammatory chemokines reported to drive cytotoxic cells in tumor microenvironment (TME), has been recently identified as a direct transcriptional target of HIF-1α. In a study by Benoit et al., the decreased ACKR2 expression observed following HIF-1α depletion was associated with an augment of Chemokine (C-C motif) ligand 5 levels in hypoxic B16F10 CM cells, providing novel evidence on how hypoxia might impair pro-inflammatory TME and hamper immune infiltration [63].

## HIF-2α-related phenotypes in hypoxia and normoxia in CM

While HIF-2α has been largely characterized in renal cell carcinoma, where it has been shown to represent a key driver of this disease, studies focused on its function in other human malignancies, including CM, are still in their infancy. At present, however, it is known that, similarly to HIF-1α, HIF-2α can regulate several pathways, thus contributing to the malignant behaviors of CM cells (Table 1).

*Angiogenesis and vasculogenic mimicry.* Unlike HIF-1α, the role of HIF-2α in regulating angiogenesis is still ambiguous and not complete clarified. As stated above, ET-1 production sustained HIF-1α/HIF-2α-mediated VEGF-A and VEGF-C expression in CM and EC, thus promoting neo-vascularization [39]. Surprisingly, and in contrast with the study by Spinella et al., a putative tumor-suppressor role for HIF-2α has been also described. In fact, stabilization of HIF-2α with the proline hydroxylases (PHD) 3 inhibitor AKB-6899 decreased angiogenesis in B16F10 CM-bearing mice and in A375 CM cell-line derived xenograft, by promoting secretion in the tumor environment of a soluble form of the VEGF receptor (VEGFR), which usually inhibits VEGF activity. On the contrary, HIF-1α accumulation and VEGF production were not affected by AKB-6899 treatment [64].

*EMT*,* metastasis and stemness.* In the last years, a number of evidence has confirmed that aberrant HIF-2α activation contributes to CM metastasis. Under hypoxia, HIF-2α promotes EMT and cell migration by enhancing the expression of specific target genes, which can be, or not, commonly regulated by HIF-1α. For instance, HIF-2α, but not HIF-1α, directly enhanced Snail expression in CM cells, fostering features associated with stemness in vitro and increasing metastatic capacity in vivo [65]. A mass spectrometry analysis of HIF-2α interactome identified specific HIF-2α interactors involved in CM development, including MITF, SRY-box transcription factor (SOX) 10, and adaptor protein-2α (AP2α). Although MITF and SOX10 were confirmed as HIF-1α partners as well, the transcription factor AP2α was not. Furthermore, HIF-2α and AP2α interaction had functional consequences for the invasive properties of CM cells since HIF-2α overexpression in AP2α-expressing cells could reduce their invasion potential [66]. On the other hand, an independent silencing of HIF-1α and HIF-2α performed in metastatic CM cells K457 by using short hairpin RNA (shRNA) identified connective tissue growth factor (CTGF), an invasion positive MMPs regulator, as a specific target of both isoforms. Inhibition of CTGF decreased invasion and migration of CM cells along with reduced MMP-9 expression [67]. shRNA constructs against HIF1α and HIF2α did not affect tumor initiation and progression in PTEN-deficient, BRAF-mutant genetically engineered mouse model of CM, but clearly abrogated metastasis at lymph node. Mechanistically, HIF-1α and HIF-2α drove CM invasion through Platelet Derived Growth Factor Receptor Alpha (PDGFRα) and Focal Adhesion Kinase (FAK)-dependent SRC activation, and by directly regulating the expression of MMPs involved in invadopodia-associated ECM degradation. Notably, HIF-1α and HIF-2α acted independently since HIF-1α positively regulated *PDGFRα*-dependent SRC activation and Metallothionein 1-MMP expression, whereas HIF-2α specifically fostered *FAK* signaling and MMP-2 expression in CM cells cultured in hypoxic conditions [68].

*Immunomodulation.* Regulatory T (Treg) cells usually prevent effective anti-tumor immune response and promote immune evasion, thus playing a crucial role in CM initiation and progression [69]). However, some Treg cells can be converted into effector T cells, which lose suppressive capacity and release pro-inflammatory cytokines to promote inflammation [70]. In this context, Treg-selective HIF-2α deletion or treatment with the HIF-2α PT2385 inhibitor reduced Treg capacity to impair the activities of effector T cells, and suppressed cells dissemination in immunogenic B16F10 CM mouse models, apparently via increasing HIF-1α expression [70]. In contrast, in a study by Doedens et al., HIF-2α, along with HIF-1α maintains the functions of CD8^+^ cytotoxic T lymphocytes (CTL) [71], which are the front-line defense in the elimination of infected or tumor cells [72]. In fact, in CTL, HIF-1α and HIF-2α sustained the expression of effector molecules, co-stimulatory receptors and key transcriptional regulators, thus hampering tumor growth in CM mouse models [71].

## HIF-α regulation in CM

Canonical-oxygen dependent negative regulation of HIF-α signaling is commonly driven by the PHD 1–3 proteins. PHDs hydroxylate two conserved proline residues in the ODD motif of HIF-α subunits, in order to promote the binding of HIF-α with the von Hippel-Lindau protein (VHL) that leads to its subsequent proteasome-dependent degradation [2]. Surprisingly, Liu et al. found that psychological stress enhanced EMT and vasculogenic mimicry in CM by reducing the ubiquitin-mediated degradation of HIF-1α through the activity of the D2 dopamine receptor (DRD2), which expression was upregulated in tumor tissues under stress condition. More precisely, DRD2 could interact with VHL and competitively inhibit the HIF-1α/VHL interaction, resulting in increased HIF-1α stability [73]. In addition, the transcriptional activity of both HIF-1α and HIF-2α is also negatively regulated through the hydroxylation of an asparagine residue in the C-TAD domain, mediated by the factor-inhibiting HIF, that blocks the HIF-α binding to CBP/p300 co-transcription factors [74]. These processes are hampered under hypoxic conditions, since HIF-α subunits accumulate and heterodimerize with HIF-1β to form a transcriptional complex that translocates into the nucleus [75].

**Fig. 2:**
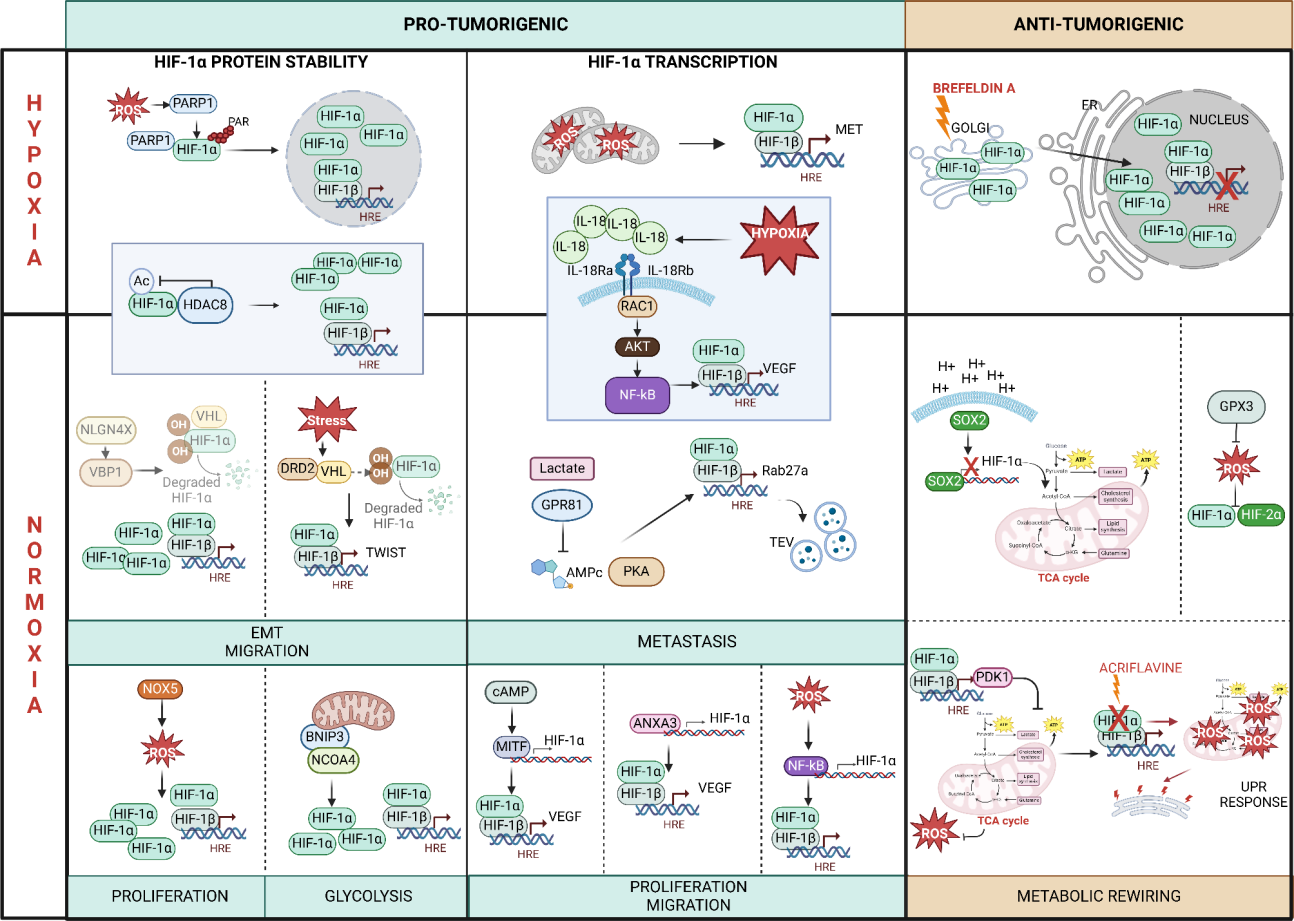
Overview of the multiple pathways impacting on HIF-1α in CM. In the upper panel, a graphic representation of the main pathways regulated by HIF-1α under hypoxic conditions (1 kPa O_2_ levels) in CM is shown. Conversely, the lower panel resumes pathways that are controlled by HIF-1α in normoxia (18 kPa O_2_ levels). Panels are divided into pro-tumorigenic (blue) and anti-tumorigenic (orange). Created in BioRender (https://BioRender.com/p73u915). Abbreviations: HIF: Hypoxia-inducible factor; ROS: Reactive oxygen species; PARP1: Poly (ADP-ribose) polymerase; PAR: Poly ADP-ribose; HDAC8: Histone deacetylase 8; IL-18R: interleukin-18 receptor; RAC1: Rac Family Small GTPase 1; NF-kB: The Nuclear Factor Kappa B; VEGF: Vascular endothelial growth factor; ER: endoplasmic reticulum; NLGN4X: Neuroligin; VBP1: VHL Binding Protein 1; VHL: Von Hippel-Lindau; DRD2: D2 dopamine receptor; GPR81: G protein-coupled receptor 81; PKA: protein kinase A; TEV: tumour-derived extracellular vesicles; SOX2: SRY-box transcription factor; TCA: Tricarboxylic acid; GPX3: Glutathione Peroxidase 3; NOX5: NADPH oxidase 5; EMT: Epithelial to mesenchymal transition; BNIP3: BCL-2 interacting protein 3; NCOA4: Nuclear Receptor Coactivator 4; MITF: Microphthalmia-associated transcription factor; ANXA3: Annexin A3; PDK1: Pyruvate dehydrogenase kinase; UPR: unfolded protein response

In addition to the canonical regulation, supplementary factors can influence HIF-1α transcription, protein stability and activity in response to hypoxia in CM (Fig. 2). Among them, poly (ADP-ribose) polymerase 1 (PARP-1) has recently emerged as a novel HIF-1α regulator in CM. Mechanistically, following incubation in a hypoxia chamber, Martí JM et al. observed an early ROS induction that led to PARP-1 activation in C8161 metastatic CM cells. Once activated, PARP-1 stabilized HIF-1α by PARylation at specific lysine and arginine residues within the C-terminus domain. Consistently, PARP-1 and HIF-1α expression were strongly associated in tissues from CM patients, thus suggesting that PARP inhibitors might be considered as a potential therapeutic approach in CM displaying HIF-1α overactivation [76]. Intriguingly, Lakhter et al. unveiled that HIF-1α could localize at Golgi compartment in CM cells cultured in hypoxia chamber. Treatment with brefeldin A, an ER-Golgi transport inhibitor, not only induced HIF-1α protein accumulation in the nucleus, but also reduced its transcriptional activity, indicating that ER-Golgi transport is essential to fine-tuning the transcriptional activity of HIF-1α [77].

In cancer, hypoxia is only one factor among several HIF-α-activating mechanisms. Interestingly, normoxic activation of HIF-1α has been detected in several cancer cell types, including CM, where contributes to the acquisition and maintenance of a malignant phenotype [78, 79] (Fig. 2). Accordingly, Martí-Díaz et al. observed that, in normoxic conditions, the inhibition of HIF-1α reduced glucose viability, impaired glucose metabolism, and suppressed CM cell survival [80]. In line with these findings, in a study by Andreucci et al., the SOX2-driven HIF-1α inhibition decreased the glycolytic activity and induced a shift towards oxidative phosphorylation in CM cells in normoxia [81].

Different non-hypoxic stimuli could enhance HIF-1α signaling, including lipopolysaccharides [81], thrombin, angiotensin I and cytokines [82]. As reported above, a study by Luo et al. showed an interplay between lactate stimulation and HIF-1α-driven CM metastasis formation in vivo. In detail, lactate could bind to G protein-coupled receptor 81 (GPR81) to decrease cAMP levels and the subsequent protein kinase A (PKA) activation, finally suppressing the ubiquitin-mediated proteolysis of HIF-1α [53].

At present, several proteins have been listed as HIF-1α non-hypoxic activators in CM. In a recent paper by Schörghofer et al., decreased amount of the adhesion molecule Neuroligin (NLGN4X) has been correlated with increased HIF-1α accumulation and migratory phenotypes in metastatic CM. The same group further demonstrated that HIF-1α regulation by NLGN4X was primarily mediated by Von Hippel-Lindau Binding Protein 1, a member of the canonical prefoldin complex [83]. In hypoxia, MITF expression was found to be suppressed following HIF-1α activation in CM [41–43]. However, in response to cAMP, MITF bound to the HIF-1α promoter to stimulate its transcriptional activity in normoxic melanocytes and CM cells [84]. Mitochondrial ROS have been involved in HIF-1α regulation in hypoxia in CM [38]. Besides mitochondria, ROS can be produced in the cytosol as well, and their accumulation can also occur in non-hypoxic conditions. For instance, cytosolic NADPH oxidase 5 (NOX5) contributed to sustain CM cell proliferation, at least in part, by generating high local concentration of ROS that in turn, regulated normoxic HIF-1α expression [85]. ROS are known to activate NF-κB [86], and NF-κB activation might enhance HIF-1α transcription under hypoxic and normoxic environments [87, 88], suggesting a potential crosstalk among ROS, NF-κB and HIF-1α. Consistent with this hypothesis, NF-κB activation triggered by ROS increased the normoxic HIF-1α activity, whereas NF-κB inhibition attenuated accumulation in CM cell lines [79]. In another study by Kim J et al., interleukin (IL)-18 signaling induced HIF-1α expression through the Rac1/NF-κB pathway in the mouse CM B16F10 cell line. Of interest, following treatment with the antioxidants *N*-acetyl-l-cysteine and pyrrolidine dithiocarbamate (PDTC), IL-18 was not able to up-regulate HIF-1α expression. Furthermore, PDTC alone, or in combination with an inactive form of Rac1, markedly reduced NF-κB activation, suggesting that once activated by IL-8 signaling, Rac1 promoted ROS generation to mediate NF-κB activation and HIF-1α expression. Even though the molecular mechanism has been investigated under normoxic conditions, hypoxic stimulation dramatically increased IL-18 and HIF-1α mRNA expression, indicating that IL-18 might perform regulatory functions in HIF-1α expression under both normoxic and hypoxic conditions [89]. Although the molecular mechanism is yet to be defined, the calcium-dependent phospholipid-binding protein annexin A3 (ANXA3) has been implicated in HIF-1α aberrant activation in CM as well. In fact, in a study by Xu et al., ANXA3 overexpression increased HIF-1α and VEGF protein expression levels [90]. In CM cells, under conditions of oxygen availability, BCL-2 interacting protein 3 (BNIP3) could act as the upstream regulator of the HIF-1α-mediated glycolytic program. More specifically, by controlling the intracellular availability of iron and the mediated autophagic degradation of ferritin, BNIP3 was found to regulate HIF-1α stability [91]. Since BNIP3 represents one of the main HIF-1α target genes, results from this study indicated that this bidirectional loop between BNIP3 and HIF-1α fostered normoxic HIF-1α-linked glycolysis and pro-tumorigenic program in CM. Finally, the histone deacetylase 8 (HDAC8) directly bound to HIF-1α to sustain the stabilization of the protein in both hypoxia and normoxia in CM [92]. On the other hand, the glutathione peroxidase 3, an enzyme involved in protecting cells from oxidative stress, and often downregulated in CM, was reported to reduce HIF-1α and HIF-2α stability and activity by inactivating ROS production [93].

## Non-coding RNAs associated to HIF-α signaling

Functional RNAs that are not translated into proteins are called non-coding RNA (ncRNA) (for review see [94]). In the last years, a growing number of studies have revealed their importance as key molecular mediators in several many critical aspects of tumor biology, including hypoxia and HIF-α signaling [95, 96].

## MicroRNAs involved in HIF-α regulation in CM

MicroRNAs (miRNAs) are small single-stranded ncRNAs whose length ranges between 18 and 25 nucleotides. MiRNAs are transcribed as pri-miRNAs by RNA polymerase II. Pri-miRNAs are then processed by the complex formed by DROSHA/DGCR8 to produce miRNA precursors (pre-miRNAs). Thereafter, pre-miRNAs are transported to the cytoplasm where they are processed by the RNase III enzyme DICER/TRBP2 to form the mature miRNA duplex. This miRNA duplex includes the two-strand pair miR-3p/miR-5p, each of which may be selected by the ARGONAUTE proteins associated with the RNA-induced silencing complex. The final single-stranded miRNA can regulate gene expression at the post-transcriptional level through fine-tuning of target protein expression. It has become increasingly clear that miRNAs deregulation is a hallmark of cancer, where they might function as oncogenes to promote tumor formation (oncomiRs), or act as tumor suppressors. Not surprisingly, miRNAs are involved in cancer development and progression, including the metastatic process and the TME remodeling (for review see [97]).

**Fig. 3:**
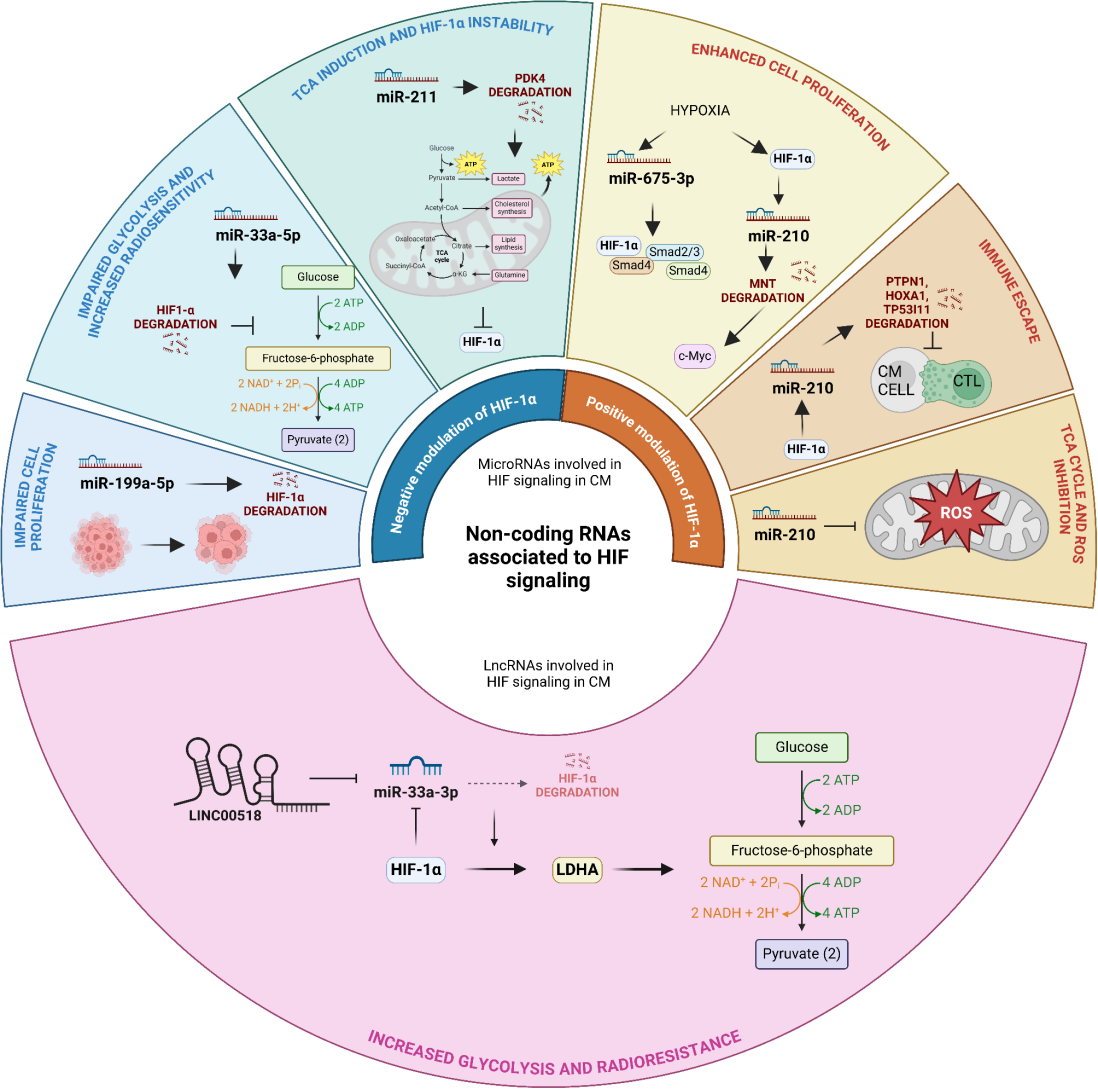
Schematic representation of non-coding RNAs involved in HIF-1α pathway regulation in CM. In the upper semicircle, a summary of the current advances about the bidirectional interactions between HIF-1α and miRNAs in CM (blue: suppressor miRNAs; orange: oncogenic miRNAs). The lower semicircle illustrates a graphic representation of the mechanism by which the lncRNA linc00518 modulates HIF-1α expression in CM. Created in BioRender (https://BioRender.com/833o824). Abbreviations: HIF: Hypoxia-inducible factor; miR: mirna; CM: cutaneous melanoma; ROS: Reactive oxygen species; MNT: MAX Network Transcriptional Repressor; CTL: cytotoxic T lymphocyte; TCA: Tricarboxylic acid; PDK4: Pyruvate Dehydrogenase Kinase 4; LDHA: Lactate dehydrogenase A; PTPN1:Protein Tyrosine Phosphatase Non-Receptor Type 1; HOXA1: Homeobox A1; TP53I11. Tumor Protein P53 Inducible Protein 11.LDHA: Lactate dehydrogenase A; PTPN1:Protein Tyrosine Phosphatase Non-Receptor Type 1; HOXA1: Homeobox A1; TP53I11. Tumor Protein P53 Inducible Protein 11

Regulation of HIF-1α on miRNAs is common during cancer initiation and progression (for review see [98]). However, recent evidence has documented complex interactions between HIF-1α and miRNAs since, besides to be directly or indirectly regulated by HIF-1α, miRNAs can modulate the expression or activity of HIF-1α as well (Fig. 3) [96, 99, 100].

*MiRNAs that negatively modulate HIF-1α.* HIF-1α was identified as a target of miR-199a-5p in several tumors, including CM. In fact, miR-199a-5p mimics impaired CM cell proliferation by decreasing HIF-1α mRNA and protein expression both in vitro and in vivo. Consistently, low expression levels of miR-199a-5p were observed in CM, particularly in tissue samples from patients with advanced tumor stage [101]. Zhou et al. reported that miR-33a expression in metastatic CM cells was also relatively low. Interestingly, when overexpressed, miR-33a inhibited HIF-1α and, consequently, reduced BRAF-mutant CM cell proliferation and invasion in vitro, and tumor growth in vivo [102]. Subsequently, Cao et al. confirmed that miR-33a-5p expression was decreased in CM cells, and that miR-33a-5p negatively regulated HIF-1α, thereby repressing glycolysis and increasing radiosensitivity of CM cells [103]. In addition to miR-199a-5p and miR-33a, Mazar et al. found that miR-211-5p indirectly reduced HIF-1α protein levels and decreased CM cell growth in hypoxia. In detail, when overexpressed in A375 BRAF-mutant CM cells, miR-211-5p improved mitochondrial respiration by targeting PDK4. On the contrary, miR-211 down-regulation led to increased PDK4 protein expression, which finally resulted in less oxidative phosphorylation, reduced mitochondrial activity and HIF-1α stabilization. Accordingly, miR-211-5p was found to be downregulated during CM progression [104]. Intriguingly, although miR-211-5p overexpression was more frequent in primary tumors respect to metastases, in a study by Moritz et al., high miR-211-5p expression in CM metastases, but not primary tumors, was associated with worse overall survival [105]. In line with these findings, miR-211-5p was upregulated in CM cells resistant to BRAFi, and involved in the early phases of resistance acquisition to targeted therapy through HIF-1α-independent mechanisms [106, 107].

*HIF-associated oncogenic miRNAs.* Among miRNAs that positively support HIF-α signaling, miR-675-3p was significantly up-regulated in CM cell lines, tissues and peripheral blood from CM patients. In vitro analyses indicated that miR-675-3p might function as a mediator of many cellular pathways in CM, since the use of a miR-675 mimic could activate TGFβ and HIF-1α signaling pathways [108]. In a subsequent study by Hwang et al., high levels of HIF-1α transcriptional activity were detected in a panel of TGFβ1 positive CM cell lines under normoxic conditions, and HIF-1α silencing downregulated several miRNAs, including miR-210, miR-218, miR-224 and miR-452 [109]. MiR-210 is a direct target of both HIF-1α and HIF-2α, and its expression has been correlated with metastasis, poor prognosis and early risk of recurrence in CM [110, 111]. HIF-1α-dependent miR-210 upregulation impaired the ability of immune system to target CM cells. Indeed, miR-210 overexpression promoted immune escape from CTL-mediated lysis by degrading protein tyrosine phosphatase non-receptor type 1, homeobox A1, and tumor protein p53 inducible protein 11 [112]. Besides to impact on immune response, miR-210 might also influence mitochondrial activity in CM. In fact, miR-210 silencing accelerated mitochondrial respiratory, thus improving ROS elimination and labelling CM cells for self-destruction, in both normoxic and hypoxic conditions [113, 114]. Therefore, miR-210 might act as a key factor in HIF-1α-mediated tumorigenesis, thus representing a good prognostic and therapeutic target in both normoxic and hypoxic CM.

At present, differently from HIF-1α, only a study by Hao et al. has focused on HIF-2α-associated miRNA in CM. More specifically, HIF-2α protein was found to mediate stemness of CM cells by regulating the miRNA-363-3p/p21 axis [115]. Nevertheless, the deeper correlation between HIF-2α and miRNA-363-3p remains to be further explored.

## Long non-coding RNAs involved in HIF-α regulation in CM

Long non-coding RNAs (lncRNAs) are defined as ncRNAs of more than 200 nucleotides with little or no coding information. LncRNAs can be located either in the nucleus or the cytoplasm, or in both compartments simultaneously, and can be classified into five categories: sense, antisense, bidirectional, intronic, and intergenic. LncRNAs have been involved in a wide range of cellular mechanisms in several tumors, including CM (for review see [94]).

To our knowledge, LINC00518 is the only lncRNA that has been characterized for its role in regulating HIF-1α expression in CM [116]. More precisely, LINC00518 sponged miR-33a-3p to increase the expression of HIF-1α, thus accelerating glycolytic metabolism and triggering radioresistance in BRAF-mutant CM cells. Additionally, HIF-1α recruited HDAC2 to the miR-33a-3p promoter region to decrease its expression. Hence, the LINC00518/miR-33a-3p/HIF-1α axis might represent an efficient target to increase the therapeutic efficiency of radiotherapy in CM [116].

## Therapeutic opportunity in CM

As mentioned above, HIF-α subunits not only play a crucial role in the response of CM cells to hypoxia but also regulate different biological processes under normoxic conditions, thus representing attractive therapeutic molecular targets. In the last years, several anticancer drugs that modulate HIF-1α expression and/or activity without directly targeting it have been described.

HIF-1α significantly impacts on CM cell metabolism as it influences the expression of different genes involved in glycolysis. Among the key effectors of HIF-1α-mediated metabolic remodeling, the 6-phosphofructo-2-kinase/fructose-2,6-biphosphatase 3 (PFKFB3) enzyme plays a crucial role in glycolysis since, once activated, it stimulates allosterically the activity of PFK-1 in BRAF-mutant CM, fostering the Warburg’s effect. Intriguingly, (2E)-3-(3-Pyridinyl)-1-(4-pyridinyl)-2-propen-1-one (3PO), an inhibitor of PFKFB3, was shown to interfere with the HIF-1α/PFKFB3/PFK-1 axis, and to induce cell cycle arrest in G1/0, apoptosis and glucose uptake reduction in BRAF-mutant A375 CM cells [117].

In a study by Furfaro et al., MeOV-1 CM cells cultured under 18 kPa O_2_ (normoxia), 5 kPa O_2_ (physioxia), and 1 kPa O_2_ (hypoxia) were treated with BRAFi. Of interest, HIF-1α expression was significantly decreased by the treatment under all O_2_ tensions tested [118]. Consistently, BRAFi vemurafenib (PLX4032) suppressed HIF-1α expression at the mRNA and protein levels in a panel of BRAF^V600^ CM cell lines cultured under standard conditions. More interestingly, HIF-1α expression was also clearly decreased early on treatment with BRAFi and restored after progression in a subset of CM biopsies. Similarly, the expression of several glycolysis-associated genes (i.e. *HK2*, *SLC2A1* and *SLC2A3*) was significantly decreased following BRAFi treatment and restored in CM patients that developed drug resistance [28], suggesting a possible involvement of HIF-1α in resistance to BRAF-targeted therapies. Under hypoxia, HIF-1α levels were decreased in CM cells exposed to PLX4032 in a cell type-dependent manner [82, 119], further suggesting that, besides to favor tumor heterogeneity, HIF-1α might influence the response to the drug treatment. Accordingly, Qui et al. demonstrated that hypoxia, by inducing MET and HGF via HIF-1α, prompted the acquisition of vemurafenib resistance in CM BRAF-mutant models both in vitro and in vivo [120]. Blocking MET signalling potentiated the antitumor effect of vemurafenib, indicating that MET inhibition might represent a potential therapeutic strategy for overcoming HIF-1α-driven PLX4032 resistance in CM patients.

In NRAS-driven CM, the growth factor receptor-binding protein 2-associated protein 2 (GAB2) has been reported to enhance tumor formation and angiogenesis by stabilizing HIF-1α at the post-transcriptional level. Histological examination of GAB2^wt^/NRAS^G12D^ xenografts showed longer vessels with markedly dilated lumina, together with increased expression of CD31, VEGFR2, VEGF, respect to NRAS^G12D^ tumors. Pharmacological inhibition of MEK significantly suppressed angiogenesis, providing evidence that HIF-1α mediated angiogenic response was dependent on RAS-RAF-MEK-ERK signaling in GAB2/NRAS-driven tumorigenesis [121].

At present, no study has evaluated the potential contribution of NF1 mutation in HIFs signalling activation in CM. Conversely, in c-Kit mutated melanocytes, HIF-1α overexpression was found to promote oncogenic transformation. Treatment with imatinib, a c-Kit inhibitor, reversed proliferation and anchorage-independent growth of melanocytes, confirming that HIF-1α induced melanocytes transformation in a c-Kit-dependent manner [122].

PTEN has been clearly identified as a potent negative regulator of angiogenesis via inhibition of HIF-1α signaling. In normoxic conditions, PTEN enhances HIF-1α ubiquitination and degradation, whereas PTEN inactivation increases the transcriptional activity of HIF-1α in hypoxia. Hence, to restore PTEN expression or activity might impair HIF-1-α-induced angiogenesis, with potential therapeutic benefit in PTEN-deficient CM. In this context, it has been proposed that the administration of plant-derived natural bioactive compounds with demethylating effects, such as phytochemicals, might favor PTEN upregulation [123].

So far, a broad spectrum of molecules of natural origin has proven to affect HIF-1α expression. Among them, isoliquiritigenin was effective in suppressing cell proliferation and in inducing apoptosis via reduced HIF-1α protein stability and mRNA expression of a number of glycolysis-related genes in B16F10 transplanted mice [124]. Vanillin was also reported to possess anti-cancer and anti-metastatic activities in CM. In particular, vanillin treatment significantly decreases HIF-1α mRNA expression by suppressing STAT3 transcriptional activity in CM cells [125]. A subsequent study by Li et al. indicated that luteolin, a natural flavonoid, exhibited a strong anti-cancer activity in CM by impairing angiogenesis and EMT both in vitro and in vivo. This effect was mainly achieved through the disruption of the HIF-1α/VEGF signaling pathway [126]. Finally, celastrol, a chemical compound extracted from the root extract of the Tripterygium Wilfordii, reduced the expression of HIF-1α mRNA by inhibiting the PI3K/Akt/mTOR signaling, leading to ROS accumulation, a decrease in that mitochondrial membrane potential that promoted cell cycle arrest and apoptosis [127].

Recent evidence has unveiled that HIF-1α has an important role in modulating recruitment and functions of immune cells to create an anti-tumorigenic microenvironment. Chromatin immunoprecipitation and luciferase reporter assay revealed that HIF-1α directly bound to a transcriptionally active HRE within the PD-L1 proximal promoter. Hence, hypoxia could modulate HIF-1α-transcriptional activity in order to increase PD-L1 expression on macrophages, dendritic cells and CM mouse models [128]. Along this line, in a study by Barsoum et al., HIF-1α induced PD-L1 expression in human breast and prostate cancer cells, as well as in mouse CM and mammary carcinoma cells, leading to resistance to CTL-mediated lysis [129]. More recently, high-throughput analyses performed in metastatic CM patients correlated hypoxia with non-response to anti-PD-1 and with immunosuppression and changes in tumor-stromal communication in TME. Intriguingly, this response was mainly orchestrated by the expression of HIF-2α in EC and fibroblasts within CM microenvironment. Indeed, HIF-2α inhibition with the small molecule PT2399, in combination with anti-PD1, delayed tumor growth in mice implanted with B16F10 murine CM cells. However, enhanced HIF-1α expression was observed in intra-tumoral EC following the anti-PD-1/PT2399 treatment. These findings suggest that the lack of HIF-2α could provoke a compensatory increase of the HIF-1α signaling to improve tumor growth control [130], thus impairing the effect of the combined anti-PD-1/HIF-2α inhibition.

In an in vivo model proposed by Wei et al., tumors form the aggressive B16F10 CM line grew significantly slower in mice lacking HIF-1α in their CD8^+^ T cells with respect to those implanted in wild-type mice. Similar effects were observed in CD8^+^ T cells treated with the HIF-1α inhibitor acriflavine alone, whereas the combinatorial treatment using acriflavine and the Treg depletion agent cytoxan further suppressed tumor development by improving CD8^+^ T cell responses [131]. Consistently, in a study by Lequez et al., the impairment of the transcriptional activity of HIF-1α significantly reduced B16F10 CM tumor growth by enhancing NK, CD4^+^, and CD8^+^ T cell infiltration. Acriflavine ameliorated the therapeutic benefit of anti-PD-1 immune checkpoint- and TRP-2 vaccine-based cancer immunotherapy [132]. Another HIF-1α inhibitor, namely IDF-11,774, also improved CD8^+^ T cell infiltration and impaired invasiveness of B16F10 CM cells in vivo by reducing the expression of the EMT markers SNAIL and N-cadherin [133]. On the other hand, dimethyloxalylglycine, a hypoxia mimetic agent, induced HIF-1α, HIF-2α and HIF-1β binding to the genes encoding the costimulatory receptors CD81, GITR, OX40 and 4-1BB, rising their expression in CD8^+^ T cells, and increasing T-cell mediated killing of CM cells in vivo. Similar effects were observed in CD8^+^ T-cells treated with the PHDs inhibitor molidustat [134]. Altogether, these results suggest that, although to target intra-tumoral hypoxia might improve the response to immunotherapy [135], HIF-α signaling activation in T-cells might further stimulate anti-tumor immunity in CM.

## Conclusion

HIF-α subunits play an essential role in cancer since they modulate the transcription of several genes involved in tumour progression and response to therapies. As we have discussed in this review, although HIF-1α and HIF-2α are closely related and share similar domain structures, it is worth noting that they are mainly non-redundant and have distinct target genes in CM. Therefore, HIF-α subunits are driver of multiple aggressive features in CM, including angiogenesis, EMT and metastasis, metabolic alterations, but also immune escape.

The pleiotropic effects of HIF-α subunits in CM suggest that inhibiting both HIF-1α and HIF-2α might offer greater therapeutic benefits respect to target either protein alone. In this setting, combination therapies that incorporate HIF-α inhibitors with existing treatments, such as BRAF-targeted therapy and/or immune checkpoint inhibitors, might provide novel therapeutic options for CM patients with recurrent or metastatic disease. At present, however, the majority of studies with molecules that inhibit HIF-α expression or promote its degradation are mostly restricted to pre-clinical models. In the future, CM humanized mouse models may allow to accurately assess the efficacy of combining HIF-α inhibitors and immunotherapy.

HIF-1α and HIF-2α expression is regulated by different pathways involving both oxygen-dependent and oxygen-independent mechanisms. While the canonical oxygen-dependent HIF-α regulation has been well described, the mechanisms by which HIF-α subunits maintain their stability under normoxic conditions in CM are still less understood, highlighting a critical area for further investigation. These observations implicate that future drug development should focus on defining additional molecular pathways that allow HIF-1α subunits to escape degradation in normoxia and elucidating how this stability might contribute to CM development and progression.

## Electronic supplementary material

Below is the link to the electronic supplementary material.

Supplementary Material 1

Supplementary Material 2

Supplementary Material 3

Supplementary Material 4

## Data Availability

No datasets were generated or analysed during the current study.

## Abbreviations

ACKR2: Atypical chemokine receptor 2
ADAM12: ADAM Metallopeptidase Domain 12
ADRP: Adipose differentiation-related protein
ANXA3: Annexin A3
AP2α: Adaptor protein-2α
AXL: AXL Receptor Tyrosine Kinase
ATP5ME: ATP Synthase Membrane Subunit E
bHLH: Basic helix-loop-helix
BIRC7: Baculoviral IAP repeat-containing protein 7
BNIP3: BCL-2 interacting protein 3
BRAFi: BRAF inhibitor
CAPN3: Calpain-3
CBP: CREB-binding protein
CM: Cutaneous melanoma
COL13A1: Collagen type XIII alpha 1 chain
CRIM1: Cysteine-rich motor neuron 1
CTGF: Connective tissue growth factor
CTL: Cytotoxic T lymphocyte
DAPK1: Death Associated Protein Kinase 1
DRD2: D2 dopamine receptor
EC: Endothelial cells
ECM: Extracellular-matrix
EMT: Epithelial to mesenchymal transition
ET-1: Endothelin-1
FABP3: Fatty acid binding protein 3
FABP7: Fatty acid binding protein 7
FAK: Focal adhesion kinase
FGF2: Fibroblast Growth Factor 2
FLNB: Filamin B
GALNT3: Polypeptide N-Acetylgalactosaminyltransferase 3
GLUT: Glucose transporters
GM3S: Ganglioside GM3 synthase
GPM6B: Glycoprotein M6B
GPR81: G protein-coupled receptor 81
HDAC: Histone deacetylase
HIF: Hypoxia-inducible factors
HK2: Hexokinase 2
HRE: Hypoxia response elements
HS3ST3A1: Heparan Sulfate-Glucosamine 3-Sulfotransferase 3A1
IL: Interleukin
ITGA3: Integrin Subunit Alpha 3
KCNMA1: Potassium calcium-activated channel subfamily M alpha 1
LDHA: Lactate dehydrogenase
lncRNA: Long non-coding RNA
miRNA: MicroRNAs
MITF: Microphthalmia-associated transcription factor
MMP: Matrix metalloproteinase
MT1A: Metallothionein 1 A
MYO1D: Myosin ID
ncRNA: Non-coding RNA
NDUFA6: NADH: Ubiquinone Oxidoreductase Subunit
NLGN4X: Neuroligin
NOX5: NADPH oxidase 5
ODD: Oxygen-dependent degradation
OXPHOS: Oxidative phosphorylation
PARP: Poly (ADP-ribose) polymerase
PAS: Per-ARNT-Sim
PDGFRα: Platelet Derived Growth Factor Receptor Alpha
PDH: Pyruvate dehydrogenase
PDK: Pyruvate dehydrogenase kinase
PDTC: Pyrrolidine dithiocarbamate
PFKFB3: 6-phosphofructo-2-kinase/fructose-2,6-biphosphatase 3
PHD: Prolyl hidroxylases
PKA: Protein kinase A
PKM2: Pyruvate kinase M2
pre-miRNA: miRNA precursor
ROR: Receptor tyrosine kinase-like orphan receptor
ROS: Reactive oxygen species
SFK: Src family kinases
shRNA: Short hairpin RNA
SOX: SRY-box transcription factor
TAD: Transactivation domain
Tβ4: Thymosin beta-4
TCA: Tricarboxylic acid
TME: Tumor microenvironment
TNFRSF14: TNF Receptor Superfamily Member 14
Treg: Regulatory T lymphocyte
TYRP1: Tyrosinase related protein 1
uPAR: Urokinase-type plasminogen activator receptor
VEGF: Vascular endothelial growth factor
VEGFR: VEGF receptor
VHL: Von Hippel-Lindau
WT: Wild type
